# Imprinting: expanding the extra-pharmacological model of psychedelic drug action to incorporate delayed influences of sets and settings

**DOI:** 10.3389/fnhum.2023.1200393

**Published:** 2023-07-18

**Authors:** Nicolas Garel, Julien Thibault Lévesque, Dasha A. Sandra, Justin Lessard-Wajcer, Elizaveta Solomonova, Michael Lifshitz, Stéphane Richard-Devantoy, Kyle T. Greenway

**Affiliations:** ^1^Department of Psychiatry, McGill University, Montreal, QC, Canada; ^2^Lady Davis Institute for Medical Research, Jewish General Hospital, Montreal, QC, Canada; ^3^Department of Psychology, McGill University, Montreal, QC, Canada; ^4^Integrated Program in Neurosciences, McGill University, Montreal, QC, Canada; ^5^Neurophilosophy Lab, Department of Philosophy, McGill University, Montreal, QC, Canada; ^6^McGill Group for Suicide Studies, Douglas Mental Health University Institute, LaSalle, QC, Canada; ^7^Department of Medicine, Centre for Psychedelic Research, Imperial College London, London, United Kingdom

**Keywords:** psychedelics, ketamine, set and setting, hallucinations, imprinting, extra-pharmacological model, dreaming, preparation

## Abstract

**Background:**

Psychedelic drug experiences are shaped by current-moment contextual factors, commonly categorized as internal (set) and external (setting). Potential influences of past environments, however, have received little attention.

**Aims:**

To investigate how previous environmental stimuli shaped the experiences of patients receiving ketamine for treatment-resistant depression (TRD), and develop the concept of “imprinting” to account for such time-lagged effects across diverse hallucinogenic drugs.

**Methods:**

Recordings of treatment sessions and phenomenological interviews from 26 participants of a clinical trial investigating serial intravenous ketamine infusions for TRD, conducted from January 2021 to August 2022, were retrospectively reviewed. A broad literature search was undertaken to identify potentially underrecognized examples of imprinting with both serotonergic and atypical psychedelics, as well as analogous cognitive processes and neural mechanisms.

**Results:**

In naturalistic single-subject experiments of a 28-year-old female and a 34-year-old male, subjective ketamine experiences were significantly altered by varying exposures to particular forms of digital media in the days preceding treatments. Higher levels of media exposure reduced the mystical/emotional qualities of subsequent psychedelic ketamine experiences, overpowering standard intention-setting practices and altering therapeutic outcomes. Qualitative data from 24 additional patients yielded eight further spontaneous reports of past environmental exposures manifesting as visual hallucinations during ketamine experiences. We identified similar examples of imprinting with diverse psychoactive drugs in past publications, including in the first-ever report of ketamine in human subjects, as well as analogous processes known to underly dreaming.

**Conclusions/interpretation:**

Past environmental exposures can significantly influence the phenomenology and therapeutic outcomes of psychedelic experiences, yet are underrecognized and understudied. To facilitate future research, we propose expanding the contextual model of psychedelic drug actions to incorporate imprinting, a novel concept that may aid clinicians, patients, and researchers to better understand psychedelic drug effects.

**Clinical trial registration:**

ClinicalTrials.gov, identifier NCT04701866.

## Introduction

Psychedelic-assisted psychotherapies are currently generating tremendous scientific, corporate, and public interest, prompting many to refer to an ongoing “second wave” of psychedelic research. Recent reviews have identified promising evidence for psychedelic therapies in depression and other psychiatric disorders (Chi and Gold, 2020; Rosenblat et al., 2022), and more than 100 psychedelic clinical trials have been registered on clinicaltrials.gov since 2007—the majority in the past six years (Kurtz et al., 2021).

Given this level of interest, understanding the effects of psychedelic drugs is of critical scientific and clinical importance. While psychedelic-induced alterations of consciousness are well known to be influenced by current-moment environments, the potential influences of *past* environments have received very little attention.

Much like serotonergic psychedelics, the N-methyl-D-aspartate (NMDA) antagonist ketamine has been shown to produce rapid and potent antidepressant effects at subanesthetic doses (McIntyre et al., 2021). Public and private ketamine services have arisen around the world and the s-enantiomer of ketamine, esketamine, has been approved as a novel treatment for Treatment-Resistant Depression (TRD) in the United-States, Canada, and Europe (Walsh et al., 2022). Although the altered states of consciousness that ketamine induce are typically referred to as “dissociative” (Schartner et al., 2017; McIntyre et al., 2021), their phenomenology and neural basis overlap significantly with serotonergic psychedelics (Studerus et al., 2010).

Similar to the serotonergic psychedelics, ketamine has been employed as an adjunct to psychotherapy in psychedelic models of care since the 1970s (Kolp et al., 2014; Greenway et al., 2020). Though psychedelic approaches to ketamine are far from universal, some recent evidence suggests that ketamine’s psychiatric benefits are at least partially mediated by its capacity to facilitate mystical-type experiences, similar to those induced by serotonergic psychedelics (Dakwar et al., 2018; Dore et al., 2019; Garel et al., 2022).

Irrespective of the substance, a key aspect of the psychedelic-assisted psychotherapy approach since at least the 1960s is the concept of “set and setting” (Leary et al., 1963). This guiding axiom describes how psychedelic experiences are shaped by current-moment “mindsets” (expectations, intentions, and personality traits) and “settings” (physical, social, and cultural environments) (Carhart-Harris et al., 2018). Optimizing sets and settings is thus a key aim of the preparation and treatment phases of psychedelic therapy, due to posited therapeutic impacts (Pahnke et al., 1970; Johnson et al., 2008). Indeed, given that psychedelic drugs have been described as “amplifiers” of contextual influences, one may expect even greater benefits with patient-tailored approaches to psychedelic therapies versus conventional psychiatric treatments (Swift et al., 2018).

How set and setting might be optimized is largely an open question and current practices vary greatly (Hartogsohn, 2017). Leading psychedelic protocols recommend that preparation include psychoeducation about drug effects, efforts to enhance therapeutic alliances, and the establishment of behavioral, psychological, and/or spiritual therapeutic intentions (Johnson et al., 2008; Watts and Luoma, 2020). Additional practices can be found in the first wave of psychedelic research; for instance, Timothy Leary (who coined the term set and setting) recommended meditation, introspection, and self-examination (Leary et al., 2007; Horowitz et al., 2018). Further distinct preparatory activities can be found in traditional psychedelic practices, including fasting, dream incubation, and sexual abstinence (Winkelman, 2021). However, in spite of decades and even centuries of accumulating knowledge, there have been few modern experimental examinations of set and setting and there is little consensus regarding best practices (Griffiths et al., 2018; Golden et al., 2022).

One reason for this lack of consensus is that psychedelic drug effects—and, by extension, the effects of preparatory practices—are heavily influenced by the sociocultural contexts in which they are embedded (Lifshitz et al., 2018). Researchers and clinicians seeking to understand and optimize psychedelic drug effects must therefore grapple with the complex interactions between a given individual and their society at large.

In Western societies, there have been major sociocultural shifts since psychedelic therapy practices were largely developed in the mid-20th century—especially regarding the quantities and patterns of media exposure. This includes much more time now being spent on personal electronic devices and less on print media or outdoors (Twenge et al., 2019), and the appearance of relatively new digital-behavioral phenomena such as “binge-watching” and “doomscrolling” (Starosta and Izydorczyk, 2020; Sharma et al., 2022).

Media exposures are known to influence conscious and unconscious minds in both immediate and delayed ways. For example, video game and television content frequently appear in mind wandering, daydreaming episodes, and dreams (Stickgold et al., 2001; Nielsen and Stenstrom, 2005; Gackenbach et al., 2011). Although dreams have long served as analogies to psychedelic experiences (Hartogsohn, 2016; Kraehenmann, 2017; Greenway et al., 2020), no studies to our knowledge have investigated whether digital media, or indeed other forms of environmental stimuli, may similarly influence later psychedelic drug experiences.

This article provides preliminary evidence from several independent lines of inquiry to argue that environmental exposures—especially digital media—can exert significant influences on subsequent psychedelic experiences, days or more later. Based on our recent clinical results from patients receiving ketamine in a psychedelic model, prior phenomenological reports of a variety of serotonergic and atypical psychedelic experiences, and research on cognitive processes underlying dreaming, we propose expanding the current model of psychedelic “set and setting” to account for such delayed influences with the concept of imprinting. We provide a theoretical framework for imprinting and discuss scientific and clinical implications.

## Materials and methods

We present qualitative and quantitative data from 26 patients who received a series of intravenous ketamine-assisted psychotherapy treatments over four weeks for TRD in the context of a clinical trial investigating the effects of music (ClinicalTrials.gov: NCT04701866). This randomized controlled trial was conducted from January 2021 to August 2022 at two McGill University hospitals in Montreal, Quebec, Canada. All ketamine infusions (0.5 mg/kg bodyweight, infused over 40 minutes) were administered with key aspects of psychedelic therapy, including preparative psychotherapy, treatment sessions that included psychological support and eyeshades, and follow-up integration therapy sessions (Johnson et al., 2008).

The study involved the structured collection of qualitative data for 26 of 32 enrolled patients, including audio recordings of ketamine treatment sessions and semi-structured phenomenological interviews conducted two weeks following the treatment course. A variety of validated scales were administered following each treatment to characterize patient experiences in this trial, including the Mystical Experience Questionnaire (MEQ) (Barrett et al., 2015), the results of which are reported for the two single-subject series. Manuscripts reporting the full trial results are currently under preparation.

We focus on two single-subject cases that involved varying quantities of exposure to specific forms of media, in addition to further examples of imprinting identified by reviewing the transcripts of treatment sessions and interviews of the 24 additional patients for whom qualitative data was collected. All patients consented to participate in this trial, approved by the research ethics boards at the Douglas Mental Health University Institute (#IUSMD-20-28) and the Jewish General Hospital (#MEO-14-2022-2854), and both single-subject participants gave additional written consent for their results to be detailed in this article. In addition to the eight-week follow-up of the clinical trial, these two patients were briefly interviewed by phone 8–12 months after the trial to discuss the long-term consequences of their experiences described below.

In the aim of identifying past unrecognized examples of imprinting, we searched the PubMed, Embase, and APA PsycInfo databases for studies published in English or French prior to October 1st, 2022 using the following query: (Psychedelic$ or Psilocybin or Lysergic acid diethylamide or LSD or Ibogaine or Mescalin$ or Dimethyltryptamine or Salvia or 3,4-Methylenedioxymethamphetamine or MDMA or Ketamine) and (Phenomen* or Hallucinat* or image$ or scene$) and (Screen$ or Television$ or Picture$ or Film$ or Movie$ or Cartoon$ or Disney or priming). Abstracts of the 2084 retrieved articles were screened by at least two authors and relevant article were retrieved. Additional articles were identified by iteratively reviewing references and conducting related web searches. A consensus amongst all authors was reached for all presented potential examples of imprinting.

## Results

### Patient 1— “hijacking”

A 28-year-old female, diagnosed with TRD following 14 years of unsuccessful pharmacotherapy and psychotherapy, initially received two ketamine infusions over one week while hospitalized for depression and suicidal ideation. She was also known for generalized anxiety disorder, type one diabetes mellitus, and a chronic neuromuscular condition, and no lifetime history of self-harm or suicide attempts During this hospitalization, the patient had nearly no access to electronic devices, including in the weeks prior to her first ketamine infusion.

The patient responded robustly to these first two ketamine treatments and described them as having many typical features of psychedelic therapy: feelings of connection, introspection, emotional processing, and mysticism. They resulted in rapid and significant improvements in depressive symptoms and suicidality, and the patient was discharged after six weeks in hospital with the plan for further infusions if necessary.

Six months later, as an outpatient enrolled in the aforementioned clinical trial, she received a course of six ketamine infusions over four weeks with the same team, a nearly identical treatment protocol, and a similar treatment setting. Despite reporting a similar *degree* of psychedelic effects, her first outpatient ketamine treatment was described as having remarkably different phenomenology. Namely, the patient reported that involuntary visual hallucinations of Disney iconography “hijacked” her experience, greatly diminishing its mystical and emotional qualities.

As demonstrated in the following verbatim quotes from the session’s recordings, the discrepancies between this treatment and the two that she received while hospitalized were a source of significant disappointment and frustration for the patient:

Patient 1: *“And then I just saw Disney stuff. I don’t want to! I didn’t want to!”*
Therapist: *“This is your mind, you can’t really control it.”*
Patient 1: *“It hijacked it! And it’s my fault for always scrolling through the ‘pins’*… *I’m just annoyed that I felt like I had the Band-Aid on. It felt like I almost ended up going to important things and then Disney frickin’ covered it up.”*

As evidenced by this excerpt, the patient readily drew a link between this treatment’s visual images of Disney characters and her previously undisclosed habit of trading commemorative Disney pins on a social media forum. She described spending approximately six hours per day on this digital activity since many years, with the notable exception of her month-long hospitalization when she received her first two ketamine infusions. Of note, she also described various Disney-themed physical objects in her home environment though precise details are not available.

**FIGURE 1:**
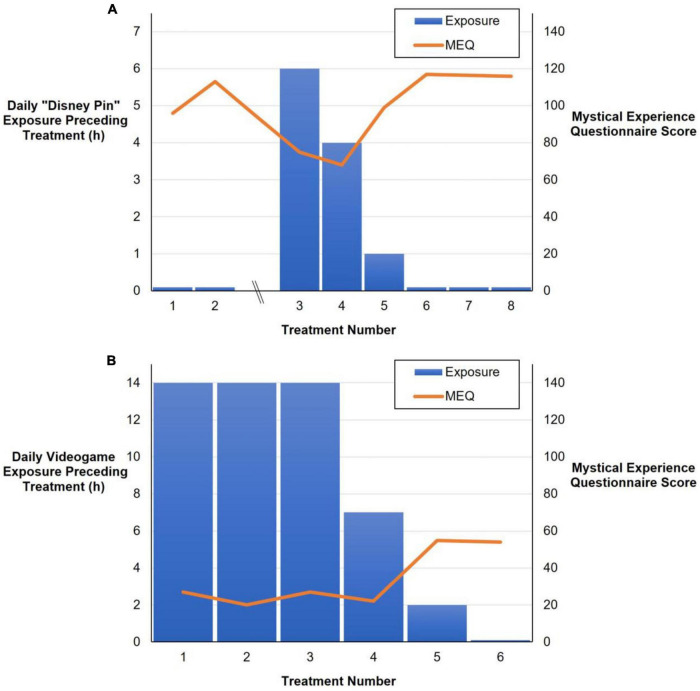
Daily hours of particular media exposure (blue bars) in the days preceding ketamine treatments and Mystical Experience Questionnaire (MEQ) total scores (orange line) for patient 1 **(A)** and patient 2 **(B)**.

Given her disappointment with this experience and its lack of emotional or mystical content, a collaborative decision was made that she would reduce her consumption of online Disney content in preparation for the subsequent ketamine treatments without any change to her physical environment. She reported thereafter reducing her daily online exposure to Disney content to approximately zero. Except for her next treatment (less than two days later), the phenomenology of her subsequent experiences over the following weeks shifted significantly, coming to resemble her initial two ketamine infusions in terms of visual phenomena and emotional/mystical content. As Figure 1 demonstrates, she reported significant increases in MEQ scores including two “complete” mystical experiences (> 60% of max score on all subscales) (Barrett et al., 2015) during these treatments—and no Disney imagery.

In her qualitative interview two weeks after the series of psychedelic ketamine treatments, the patient described an important insight resulting from the manipulation of her media habits:

“*Well, before the ketamine, I was shopping for Disney pins, like, a lot of the day, and then I felt like the first treatment was ruined because I just saw Disney and stuff, and now I’m aware when I’m doing that to numb myself, basically, and to cope, but I’m not going to lie, I’m doing it right now. But at least when I’m doing it, I know*.”

As described, the patient reported resuming her daily habit of Disney pin trading after her final ketamine treatment, albeit at a markedly reduced quantity of about one hour per day. When contacted one year following the ketamine treatments, the patient reported that this significant behavioural change persisted. Although the overall benefits of ketamine against her anxiety, depression, and suicidality were modest and short-lived, her insight regarding the effects of digital media on her mind was described as profound and lasting.

### Patient 2— “a pixelated consciousness”

A 34-year old man with TRD, in addition to morbid obesity and obstructive sleep apnea, received a series of six ketamine infusions over four weeks as an outpatient after 18 years of poor responses to numerous medications and psychotherapies. On evaluation, he described spending nearly all his waking hours outside of work playing various video games, regularly up to 16 h per day.

This patient’s first three ketamine experiences were characterized by vivid visual hallucinations described as “videogame-like” in both content and form. I.e., he reported that most of his time during the infusion was spent reliving recent game experiences and he described “pixelated” complex hallucinations that strongly resembled the aesthetic of video games like Minecraft, which he had played frequently in the days preceding the treatment sessions. He summed up his experiences as evidence that he had “a pixelated consciousness”.

Unlike the first patient, this patient was not immediately distressed by these experiences. He instead described them as “fun”—much like his experience of the videogames themselves, which he recognized as their likely source. However, like the first patient, he spontaneously drew links between these experiences and negative aspects of his lifestyle, stating: “…all I do is distract myself, play, and try to have fun.” He did not attribute significant meaning to these three ketamine experiences, and his MEQ scores were low throughout.

Like the first patient, this second patient and his therapists decided to experiment with reducing his video game playing to check if the subsequent ketamine experiences might become more personally meaningful and/or mystical-like, and ultimately more beneficial. He thus reduced his consumption by roughly half, for the four days prior to his fourth treatment. The result was a more emotionally intense ketamine experience that featured some video game imagery, but also marked feelings of grief related to past relationships. I.e., the patient reported vividly re-experiencing a series of events that led to the end of a friendship, which evoked strong feelings of shame and regret. The MEQ score remained low.

Despite this fourth treatment being more psychologically challenging, the patient was encouraged by its greater degree of meaningful autobiographical content and agreed to further reduce his video game exposure in preparation for the two remaining ketamine experiences. Thus, for the first time in roughly 20 years, he entirely stopped playing videos for multiple days, instead spending time with family and friends. Subsequently, his fifth and sixth ketamine experiences differed dramatically from the prior infusions, being characterized by intense visual hallucinations that reflected prominent themes of nature and his relationships which were no longer reported to be videogame-like or pixelated in form. His MEQ scores for these treatments roughly doubled.

On follow-up, eight months later, the patient reported significant levels of video game playing but also active engagement in weekly psychodynamic psychotherapy (that had been initiated alongside the ketamine treatments), persistent reductions in symptoms of depression and anxiety, and an increased “interest in [his] subconscious mind”.

### Additional spontaneous patient reports of imprinting

In addition to these cases, which were exceptional in terms of relatively extreme quantities of media exposure, a review of transcripts from 24 patients receiving the same ketamine treatment protocol yielded further examples of imprinting from eight other patients. These ten patients with unipolar or bipolar TRD ranged in ages from 27 to 63 and were characterized by substantial psychiatric comorbidity and chronicity. Their sociodemographic and baseline characteristics are provided in Supplementary Table 1 of the Supplementary material.

The supporting patient quotes are summarized in Table 1 with more detailed transcripts provided in Supplementary Table 2. All instances were defined by spontaneous descriptions of (mostly visual) hallucinations during ketamine experiences which vividly reflected environmental exposures of the preceding days. The majority of these exposures were digital in nature, with the exception of three examples of imprinting from recent real-life environments: a museum visit, time in nature, and a visit to a city.

**TABLE 1:** Examples of imprinting from the excerpts of eight different patients describing their ketamine treatment experiences.

Recent exposure	Supporting quote
Video	*“I was on the outside. I was looking down; I could see the set from the top. And they were shooting scenes of Handmaid’s tale*… *It’s a TV show that I’m watching right now.”*
Video	*“It was really beautiful, but there was one strange thing. I kept seeing faces from that show my sister loves*… *She always makes me watch it.”*
Video	*“I did see myself as a fish*… *Yesterday I watched ‘Luca’, which is a sea monster movie”*
Video	*“I just felt like I was in a pod in space and I was thinking “Oh, it’s like that movie*… *that I just watched.”*
Video	*“It was a moving performance. I love dance and I always watch ballet videos on my cell phone. It was like being in a show.”*
Countryside	*“Well, in my head I saw the country environment again, the fields*… *I also hear the sounds of little birds, the same then when I was lying in my bed in my trailer yesterday.”*
City	*“We were in colors, shapes, much more urban*… *That’s because we talked a lot about urbanism in the car on the way there, too.”*
Museum	*“I was definitely in the museum, again, but.in the paintings*… *I was in the art.”*

## Discussion

### Imprinting and the extra-pharmacological model

The extra-pharmacological model of psychedelics posits that drug effects are strongly influenced by the immediate environment, i.e., set and setting (Carhart-Harris and Nutt, 2017; Lifshitz et al., 2018). In this article, we present recent evidence from two single-subject case series and eight additional patients suggesting that environmental exposures can also exert pronounced delayed effects on drug phenomenology.

Both single-subject series above were notable for the quantity of daily exposure to particular forms of digital media, as well as their impact on ketamine phenomenology and therapeutic effects. The first case resembled an n-of-1 trial of A-B-A design (Lillie et al., 2011)—a pragmatic research design that has been recommended for studying psychedelic drug effects (Carhart-Harris et al., 2022)—where both exposure to Disney social media content and its subsequent manifestations in psychedelic experiences were absent, then present, then again absent across eight ketamine sessions over six months.

In the second single-subject series, video game-playing was gradually decreased over several weeks rather than abruptly stopped, with the results suggesting a potential “dose-response relationship”. I.e., a reduction in exposure to video games by approximately 50% reduced their impact on visual hallucination content and form, and several days of abstention produced two experiences free of videogame imagery with significantly increased MEQ scores. The findings of these two single-subject series led to the review of other patient transcripts and the identification of additional examples of imprinting where both the quantities of identified environmental exposures and their manifestations were more subtle.

**FIGURE 2:**
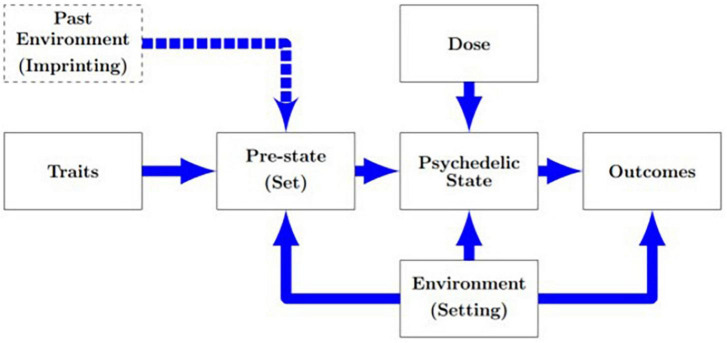
Proposed modification of the extra-pharmacological model of psychedelic drug actions (Carhart-Harris and Nutt, 2017) to incorporate imprinting effects. I.e., that in addition to current-moment environments, past sensorial exposures exert time-lagged influences on (mind)sets and psychedelic drug experiences.

Based on these results, we propose expanding the current extra-pharmacological model of psychedelic drug effects to account for delayed environmental influences with the novel concept of imprinting (Figure 2). We define imprinting as a phenomenon whereby environmental exposures prior to psychedelic sessions involuntarily and spontaneously manifest in the content and/or form of the perceptual changes of the experiences. All of the above examples of imprinting were only, or primarily, visual hallucinations, which could be described with a variety of existing terms including closed-eyes visuals, complex hallucinations, or pseudo-hallucinations (given that they were not confused with reality) (Blom, 2010). In the following section, we compare imprinting to related but distinct processes.

### Distinctions between imprinting and prior concepts

The concept of imprinting has analogies to other previously described phenomena such as priming and suggestion, which have been used in hypnosis and previous psychedelic therapies (Levine and Ludwig, 1966; Oakley and Halligan, 2013). Hypnotic suggestions are defined as declarations made to induce, or made during, hypnotic states that prescribe changes in behavior, cognition or perceptual experience that do not require volitional engagement of the participant to occur (Oakley and Halligan, 2013). Suggestion is an important comparator to imprinting given that it also occurs without voluntary engagement and because increased suggestibility has been demonstrated both in hypnotic states and during the acute effects of a wide variety of psychoactive drugs, including the serotonergic psychedelic LSD, cannabis, nitrous oxide, and ketamine (at least in some individuals) (Kelly et al., 1978; Whalley and Brooks, 2009; Carhart-Harris et al., 2015; Patterson et al., 2018). Indeed, hypnosis and suggestion have been explicitly utilized to shape psychedelic experiences to increase therapeutic benefits in an approach known as “hypnodelic therapy”, first described in the first wave of psychedelic research (Levine and Ludwig, 1966).

A major difference between imprinting versus priming or suggestion is that, in the case of our examples, the stimuli were encountered naturalistically and were not intended nor expected to be imprinted by patient or therapists. Expectancy, therefore, plays a less important role in imprinting relative to hypnotic suggestion. We also emphasize that imprinting takes place well before the experience of an altered state consciousness (drug- or hypnosis-induced). I.e., in contrast to hypnotic suggestion that generally results from immediate-environment *external* stimuli (Oakley and Halligan, 2013), imprinting is the product of past stimuli having been *internalized*—or imprinted, in other words.

Another concept from the psychedelic literature that resembles imprinting is that of “eidetic images”, or “eidetic memory images”: images “seen” with eyes closed during psychedelic experiences that are distinguished by their vividness and even photo- or film-like qualities (Masters and Houston, 2000). These terms are not commonly used at present but were discussed at length in the landmark 1966 text, *The Varieties of the Psychedelic Experience*, which defined them as images of persons, animals, architecture, and landscapes “previously recorded by the brain or, in some cases, possibly a part of the phylogenetic (or “Racial”) inheritance” (Masters and Houston, 2000). We distinguish eidetic images from imprinting in that the former are often fantastical amalgamations of multiple memories which are understood to be abstract and symbolic in nature (Masters and Houston, 2000), rather than straightforward manifestations of identifiable environmental exposures in psychedelic hallucination form or content.

The closest concept to imprinting, to our knowledge, is the uncommon pathological condition known as palinopsia, which refers to the persistence or recurrence of visual images after the stimulus has been removed and has two distinct subtypes (Gersztenkorn and Lee, 2015). Illusory palinopsia refers to indistinct images occurring in the same location of the visual field as an external stimulus, influenced by ambient light and motion, similar to physiological afterimages. Hallucinatory palinopsia refers to previously observed visual images or short scenes being visually hallucinated with remarkable fidelity and clarity for seconds, minutes, hours, or even days.

Although palinopsia overlaps significantly with imprinting and there are possible neural commonalities, there are also important differences. For one, hallucinatory palinopsia has never been associated with psychedelic drugs, and illusory palinopsia has only been considered in the context of post-acute psychedelic effects like hallucinogen persisting perception disorder (Gersztenkorn and Lee, 2015; Schimansky et al., 2022). Rather, palinopsia most commonly arises from posterior cortical lesions, seizures, and other central nervous system pathologies. Secondly, we define imprinting as a *process* and hypothesize that the underlying factors are identifiable and measurable, as discussed below. In contrast, palinopsia is a *symptom*, and the factors influencing the content of hallucinatory palinopsia are not known. Thirdly, and crucially, we provide examples of imprinting that go beyond re-experiencing certain images or scenes as they were previously encountered, but instead include the apparent generation of novel content whose *form* resembles previous environmental exposures. For instance, our second patient described a series of visual hallucinations characterized by unfamiliar content but a familiar “pixelated” form that strongly resembled a particular videogame’s aesthetic.

For these reasons, we believe that imprinting is a phenomenon distinct from suggestive priming, eidetic images, and palinopsia. Rather, as discussed below, we propose that dreaming as the most analogous process.

### Unimportant or underrecognized? Further evidence for imprinting with ketamine

Despite decades to centuries of research on psychedelic drug phenomenology, this report is, to our knowledge, the first explicit description of imprinting effects. This raises the question: is this effect unimportant, or has it been underrecognized? One argument in support of the latter is that our psychedelic-ketamine protocol has unique aspects that facilitated the recognition of imprinting effects.

Most psychedelic ketamine or serotonergic-psychedelic protocols in the clinical literature involve only one or two dosing sessions (Griffiths et al., 2018; Watts and Luoma, 2020; Carhart-Harris et al., 2021). In contrast, our protocol employed at least six ketamine treatments administered over at least four weeks, yielding roughly the same amount of time in an altered state of consciousness as two doses of psilocybin, but more spread out in time. While briefer but more frequent treatments may have disadvantages—for instance, requiring more patient visits—this treatment rhythm permitted the manipulation of media exposure, and the relatively long active treatment length aided both patients and clinicians in observing links between recent environmental exposures and a later treatment’s phenomenology. Indeed, single-subject experiments may be particularly promising approaches for the study of contextual factors in psychedelic therapy for much the same reasons (Lillie et al., 2011; Carhart-Harris et al., 2022).

Once aware of imprinting effects, we were able to retrospectively identify numerous examples in the phenomenological reports of our patients as well as unrecognized examples in the broader literature. To take one striking example, the first-ever report on the effects of ketamine in human subjects, published in 1965, described patient experiences that are consistent with imprinting:

“*At times some of the subjects had vivid dreamlike experiences or frank hallucinations. Some of these involved the recall of television programs or motion pictures seen a few days before*… *Some of these phenomena were so real that the subjects could not be certain that they had not actually occurred.”* (Domino et al., 1965)

Further, therapeutic guidance for ketamine-assisted psychotherapy implicitly suggests that at least some therapists have developed an awareness of imprinting and its therapeutic importance. In a 2014 publication, one of the pioneers of this approach, Eli Kolp, “advise[d] de-stressing the mind by limiting screen time beyond that which is required for each individual participant’s employment” (no more than two hours per day) in order to increase the likelihood of “transcendental experiences.” (Kolp et al., 2014). Although not directly stated as such, this advice is consistent with minimizing imprinting by digital media in order to promote more meaningful ketamine experiences, in perfect alignment with our two single-subject studies.

### Is imprinting unique to ketamine?

Evidence of imprinting can also be found in the psychedelic literature beyond ketamine. One of the first modern psychedelic phenomenological studies, exploring the experiences of 15 volunteers receiving psilocybin, identified nine broad categories of experiences (Turton et al., 2015). One of these categories, “Effect of Memories”, was defined as “recent memories influencing the contents of their experience, similar to the influence of recent thoughts and experiences on dream content.” For example, one participant reported: “I’ve just been on holiday in Tunisia… [I] kept seeing Tunisian-Moroccan imagery in my mind.” Three of 15 volunteers reported such effects which, despite being labeled as memories, may well be better conceptualized as imprinting. I.e., the example of Tunisian-Moroccan imagery was not described as remembering or reliving, but rather the spontaneous appearance of related visual hallucinations.

Another potential example of imprinting can be found in one of the definitive texts on the phenomenology of ayahuasca (Shanon, 2003, p. 96), a serotonergic psychedelic brew. Based on the authors’ extensive experience, this work proposes five “styles” of ayahuasca experiences of which the first is reminiscent of our first single-subject’s experience of Disney imagery:

*“Even without being asked about the style of the Ayahuasca visualizations, informants mentioned that what they saw resembled cartoons and animated movies similar to those encountered in pop art. Quite a few indicated that the visions reminded them of Disney-like designs.”*

In addition to these ayahuasca visions, similar descriptions of Disney iconography have been reported with LSD and other classical psychedelics (Masters and Houston, 2000). These accounts could simply reflect Disney being used as a shared cultural reference point or, conversely, that psychedelic drug experiences were shaped by past Disney imagery exposure as was the case for our first patient above.

Another suggestive example of imprinting comes from a phenomenological study of users of ibogaine, a hallucinogenic substance that has also received attention for therapeutic applications (Heink et al., 2017). Forty-six percent of 27 participants undergoing facilitated ibogaine treatment ceremonies reported prominent visual hallucinations of “television screens” during their experiences. Although the details of these hallucinations of screens were not reported, this phenomenology contrasts significantly with ethnographic reports of ibogaine ceremonies in Africa’s Bwiti communities, which are generally notable for movement within landscapes and interrogatory verbal exchanges with ancestors and archetypal beings (Fernandez, 2020). We propose that this discrepancy may well reflect imprinting of television screen exposure in the survey responders, a population consisting almost entirely (96%) of people who identified as White/Caucasian/European (Heink et al., 2017).

Outside of the academic literature, other probable examples of imprinting effects can be found in online accounts of drug experiences. For instance, although 3,4-Methylenedioxymethamphetamine (MDMA) is not typically associated with prominent visual hallucinations, one online “phenomenological inventory” described a variety of the author’s closed-eye visuals (Ball, 2018). Several such descriptions are strongly suggestive of imprinting effects, including a vivid film-like hallucination of John F. Kennedy’s assassination and a hallucination of a hand changing the “visual scene” by “swiping left”, an experience the author describes as “…clearly related to using a touch-screen mobile device”.

### Dreaming as a mechanistic model for imprinting

Dreams have long served as analogies to serotonergic psychedelic and ketamine experiences (e.g., “ketamine dreams”) due to both phenomenological and neural overlap (Hartogsohn, 2017; Kraehenmann, 2017). Indeed, one of the first names proposed for serotonergic psychedelics was “oneirogens”—drugs that produce dreams—a label that has also been proposed for a variety of other psychedelic drugs (Goutarel et al., 1993; Greenway et al., 2020). Perhaps unsurprisingly then, analogous processes to imprinting can be found in the study of dreaming.

A wide body of literature supports the popular notion that dream content often reflects recent environmental exposures, via a process known as dream incorporation. Children’s nightmares for example, shifted from “bogeyman” content in the 1920s to film characters in the 90s, with video game imagery arising in the early 2000s (Schredl et al., 2008). Dream incorporation has been divided into two types: “day residue”, a term coined by Freud in 1898 referring to memories from the previous day appearing in dreams (Strachey and Freud, 2010), and the “dream-lag effect”, whereby dream content is influenced by stimuli from days or even years before (Nielsen and Powell, 1992; Stenstrom et al., 2012).

The day residue effect has been robustly documented in observational and experimental studies of both night-time dreams and hypnagogic hallucinations (Nielsen, 2017). One notable study even provided evidence that this effect does not require conscious memory formation: exposure to the video game Tetris led to frequent reports of Tetris-like shapes in the hypnagogic visuals of temporal lobe amnesiacs lacking conscious memories of the intervention, much like healthy controls (Stickgold et al., 2000).

In addition to such direct effects, exposure to specific forms of media can also exert more subtle and time-lagged influences (Solomonova et al., 2015). In one study, exposure to a virtual reality flying-based game not only resulted in dreams of that game’s content over the next week, but also increased the frequency of a broader variety of flying dreams (Picard-Deland et al., 2020). Indeed, beyond dream *content*, the *form* of dreams can be markedly influenced by even distant media exposure—e.g., true greyscale dreams occur almost exclusively in people with significant and early histories of black-and-white media exposure (Murzyn, 2008).

Day residue typically consists of memory elements of banal but potentially sensorily salient experiences, typically rated by dreamers themselves as personally unimportant (Vallat et al., 2017). On the other hand, the dream-lag effect more often incorporates personally relevant details of lived experiences (van Rijn et al., 2015). In hypnagogic experiences, for example, references to films have manifested due to both day residues and remote memories, dating back days or even weeks (Stenstrom et al., 2012).

In other words, the content, themes, and aesthetic qualities of dreams are known to be shaped by recent and distant environmental stimuli in a variety of ways, and similar processes may be observed in the various examples of imprinting provided above. For instance, video game exposure leading to “pixelated” forms of visual hallucinations in the second single-subject patient resembles the way that past exposure to black and white media can result in grayscale dreams. Similarly, repeated and early exposure to Disney iconography may partially explain why serotonergic psychedelic experiences are commonly Disney-like (Masters and Houston, 2000; Shanon, 2003), regardless of recent exposure.

### Factors influencing imprinting

Based on the scientific literature of dream incorporation, and memory formation more broadly, we provide preliminary hypotheses regarding the factors that may influence the intensity and frequency of imprinting following a particular exposure (Table 2).

**TABLE 2:** Hypothesized factors influencing the strength and nature of imprinting effects for a given stimulus.

Factor	Relationship to increased imprinting
Distance in time	More recent stimulus
Duration/ Repetition	Greater duration and repetition of stimulus
Homogeneity	Greater homogeneity of stimulus
Form/Intensity	More information-rich and immersive stimuli, particularly audiovisual electronic media
Emotional arousal/Valence	Greater arousal associated with stimulus

As is the case for dream incorporation, memory, and priming effects of perceptual stimuli on subsequent stimuli, we hypothesize that imprinting effects are most likely to result from recent and prolonged exposure to a homogenous stimulus (Lavoie and O’Connor, 2013; Solomonova et al., 2015; Pearson, 2019). This is evident in both single-subject series above, where significant imprinting resulted from 6 to 18 h of daily exposure to one particular form of media, and then faded within days of that exposure being reduced or removed.

Moreover, the form/intensity of a given exposure likely influences imprinting; indeed, nearly all our examples involved visual phenomena attributed to forms of electronic media exposures. This agrees with evidence from dreaming that a stimulus’s information density and immersiveness influence its impact. For example, an experimental study found that high-fidelity video goggles with surround-sound headphones exerted greater influences on subsequent dream content than the same content delivered by lower quality, less immersive equipment (Gackenbach et al., 2011). Similarly, in the psychedelic literature, one whole-brain magnetoencephalography study found that video exposure may be uniquely powerful at “driving” neurodynamics, relative to music or the absence of external audiovisual stimuli, as reflected in brain entropy changes (Mediano et al., 2020).

An alternative possibility is that our examples of imprinting from digital media were also particularly salient—and thus particularly likely to be reported and recognized—because the imprinted content clashed with expectations for the ketamine experiences. E.g., images of Disney cartoons were interpreted as “hijacking” the experience for our first single-subject series given her expectations for mystical-type experiences.

Finally, experiences associated with greater emotional arousal and non-neutral valence are more likely to lead to dream incorporation (de Koninck and Koulack, 1975), as well as memory formation (Lavoie and O’Connor, 2013). This finding is likely equally applicable to imprinting; most of our examples involved stimuli that evoked positive emotions. On the other hand, this apparent relationship may simply reflect the fact that pleasurable media are more likely to be consumed in greater quantities.

### Imprinting, predictive processing, and the anarchic brain

The phenomenon of imprinting is harmonious with—and provides some support for—the leading neural model of psychedelic drug actions: the relaxed beliefs under psychedelics (REBUS) model (Carhart-Harris and Friston, 2019). REBUS postulates that psychedelic drugs weaken the hierarchical control of high-level processes over neural information transmission. Constraints on lower-level neural systems are thus decreased, yielding an increase in bottom-up signaling, potentially due to disruption of the brain’s default mode network (DMN).

Within the REBUS framework, imprinting may be understood as the process by which recent and prolonged (low-level) environmental exposures produce overweighted intermediate-level perceptual priors (i.e., imprints). Under the effects of psychedelics, these imprints shape the drug-increased sensory prediction errors resulting from reduced higher-level constraints, and thereby acutely manifest in conscious awareness.

To take the example of the second patient described above, near-constant exposure to pixelated videogame imagery may have temporarily created a strong attractor for the brain to interpret visual stimuli in pixelated patterns. Under the effects of ketamine, this previously inappreciable overweighted prior (imprint) of pixelation now vividly manifests as visual Minecraft-like hallucinations. Over the subsequent weeks, with reduced videogame exposure, the “pixelated prior” gradually fades in intensity, resulting in weaker effects on prediction errors and ketamine phenomenology. This fading allows for other intermediate-level priors (like past emotional experiences) to more strongly shape the perceptual prediction errors during the ketamine experience—leading to, in this case, more meaningful and mystical experiences. Such mechanisms could be investigated by neuroimaging experiments designed, for instance, to quantify the effects of previous environmental exposures on brain entropy (Mediano et al., 2020) or on resting-state functional connectivity (McCulloch et al., 2022).

Nearly all of our identified examples of imprinting are visual, which is congruent with the DMN being particularly involved in contextualizing visual processing (Huang and Sereno, 2013). Further, the fact that imprinted content arises from prior environmental exposures adds support to the REBUS model’s emphasis on psychedelic drug actions on intermediate and high neural levels, rather than lower levels such as those involved with current-moment visual perception.

### Clinical and scientific implications

We have provided preliminary examples across a variety of hallucinogenic drugs where the imprinting of various environmental exposures influenced later drug experiences in a delayed manner, including two examples with notable therapeutic consequences. In these single-subject series, reducing digital media exposures in preparation for subsequent ketamine treatments led to experiences that were more autobiographical and more mystical-like, and experienced as more beneficial—in agreement with research suggesting greater benefits with mystical or “emotional breakthrough” psychedelic experiences (Johnson et al., 2008; Dakwar et al., 2018; Griffiths et al., 2018). Accordingly, we believe that the concept of imprinting has utility for psychedelic-assisted therapy, particularly in terms of optimizing preparatory practices.

Despite a general consensus regarding some goals of preparation—to increase the likelihood of safe and beneficial psychedelic experiences (Johnson et al., 2008; Watts and Luoma, 2020)—actual practices vary greatly. Our results suggest that, when establishing therapeutic intentions prior to a psychedelic experience, it is vital to consider how behavioural patterns may support or interfere with those intentions. Excessive consumption of digital entertainment may be imprinted such that psychedelic experiences are more reflective of one’s media habits than one’s lived experiences or relationships, regardless of underlying intentions.

Our results provide some support for the benefits of preparatory practices like meditation and introspection that have been recommended by experts for decades (Leary et al., 1963; Kolp et al., 2014). In addition to direct benefits, engaging in these activities may lead to reduced time spent on countertherapeutic activities like excessive digital media consumption. As an illustration, in the only modern psychedelic study to experimentally manipulate preparatory practices to our knowledge, 50 healthy participants were randomized to receive either standard or high levels of spiritual support to facilitate the development of spiritual practices like meditation during a psilocybin treatment protocol (Griffiths et al., 2018). The group receiving additional support engaged in greater amounts of meditation practice, and reported psilocybin experiences that were somewhat more mystical and emotional (as evidenced by significantly greater rates of crying during the treatment session).

This study’s findings may not only reflect that the high-support group dedicated *more* time to spiritual practices, but also that they likely spent *less* time on digital media. Indeed, in our two single-subject series, psychedelic experiences with greater emotional and mystical content resulted from simply less time spent on digital media without any reported increase in time spent on spiritual practices. To evaluate this possibility, future studies should examine how various psychedelic therapy preparation practices influence the ways in which subjects spend their time prior to psychedelic experiences, including both increases in recommended activities as well as corresponding decreases in others.

Additionally, the concept of imprinting offers a useful lens for both therapists and patients to make sense of the phenomenology of drug experiences. A given visual hallucination may be understood as an abstract representation of the unconscious mind or as evidence of associated personal significance, as prevailing wisdom suggests (Masters and Houston, 2000; Kometer and Vollenweider, 2016). Alternatively, it may also be understood to have resulted from imprinting of a particular environmental exposure, owing more to the quantity and nature of that exposure than its intrinsic meaning.

These various contributors to psychedelic drug phenomenology are, of course, not distinct, but are rather intwined within an individual’s life experiences and sociocultural contexts (Lifshitz et al., 2018). Recent and childhood exposures to religious symbols and rituals, for example, may contribute to spiritual visions during subsequent psychedelic experiences through a complex interplay of unconscious symbolism, personal meaning, and imprinting. Such imprinting effects may be most likely to be enduring when they occur during one’s formative years, as the example of childhood black-and-white media shaping the aesthetic of dreams decades later would suggest (Murzyn, 2008).

Although interpreting psychedelic experiences through the lens of imprinting may lack in romanticism, it may also lead to important insights and or behavioural changes as our first case suggests. Indeed, this case provides very preliminary evidence that psychedelic-assisted psychotherapies may eventually find novel indications as treatments of internet gaming disorder and related conditions by making the psychological consequences of excessive media habits readily apparent.

Finally, we suggest that imprinting effects may not be limited to external stimuli but may also extend to internal stimuli like memories and imagination. In terms of brain activity, visual imagery produced by imagination can be seen as analogous to a weak form of visual perception: both involve similar patterns of neural activation in similar areas of the brain including the visual cortex, both can undergo associative learning, both can prime the subsequent interpretation of stimuli, and so on (Pearson, 2019). It is thus possible that imagining or remembering a certain image or scene may have similar, if somewhat weaker, imprinting effects relative to visually observing that image.

This may help explain why psychedelic therapists frequently report that the most important content for a particular patient tends to emerge spontaneously during psychedelic experiences (Johnson et al., 2008; Kolp et al., 2014; Mithoefer, 2017). For instance, patients with post-traumatic stress disorder or significant distress related to terminal illnesses will, almost by definition, spend significant quantities of time imagining or reliving distressing images related to these conditions. This internal exposure may serve to imprint the distressing content on the mind, such that it readily manifests during psychedelic experiences, much like repeatedly watching a related video might.

## Limitations

In this preliminary report, multiple limitations warrant consideration. This data presented were not initially collected with the intent of studying the concept of imprinting, which instead arose from our observations of patient experiences and clinical outcomes. On the upside, this means that the patterns of phenomenology we observed are unlikely to have occurred solely due to unintentionally suggestive interactions. However, the small sample size, the absence of controlled environmental exposures, and the lack of systematic or prospective information to corroborate and quantify patient reports of media habits, such as daily diaries, are all important limitations. Additionally, the imprinting outcomes were not pre-registered, and the single-subject-series were not true n-of-1 trials in that they were not designed before the intervention began (Lillie et al., 2011), but rather undertaken during naturistic collaboration between patients and clinicians. Finally, although we have identified probable examples of imprinting with multiple psychedelic drugs in diverse contexts, we are unable to determine the prevalence and magnitude of these effects or the influence of dosing contexts, such as the presence or absence of eyeshades.

## Conclusion

In this article, we have presented preliminary empirical and theoretical evidence suggesting that environmental stimuli can exert delayed and often underrecognized influences on psychedelic drug phenomenology. These effects may be subtle, or of sufficient intensity as to alter the potential benefits of the experiences (e.g., by “hijacking” them), thereby warranting consideration and study.

We introduce the concept of imprinting to account for these delayed effects and provide evidence that they may arise with a wide variety of hallucination-inducing drugs. Based on analogous cognitive processes, including from the science of dream phenomenology, we propose several probable factors that may underlie such imprinting effects from a given exposure. Future research is needed to confirm these factors, to determine whether internal stimuli (e.g., imagined images) have similar effects, and to more broadly assess the neural mechanisms, prevalence, intensity, and clinical impacts of imprinting across various psychoactive drugs.

These results have potential consequences for research and clinical practice. We suggest that future psychedelic studies examining set and setting should not focus only on immediate treatment environments. Rather, potential influences of recent and past environmental exposures require investigation and consideration, especially in populations where there may be excessive use of digital media. Further phenomenological studies are also needed to better understand, for instance, whether the common references to Disney during psychedelic experiences reflect imprinted visual exposures, or rather shared cultural references (Masters and Houston, 2000; Shanon, 2003).

For clinicians, we recommend routine consideration of how a patient’s behavioural habits may support or undermine their hopes and desires for psychedelic experiences. Excessive media exposure, as with our cases, can exert countertherapeutic imprinting effects that may well overpower intentions for personally-significant or mystical-like psychedelic experiences. Activities like meditation or introspection may therefore not only yield direct psychospiritual benefits, as experts have long recommended, but also indirect benefits via reduced countertherapeutic imprinting. On the other hand, as digital tools increasingly find roles in psychedelic-assisted psychotherapies, immersive technologies like virtual reality may well be used to shape subsequent psychedelic experiences by imprinting desired themes or images on participants’ minds in the hours-days leading up to their drug sessions.

Lastly, we suggest that it is insufficient for patients and therapists to consider only the symbolic or personal associations of particular hallucinations, nor only immediate environmental influences. Rather, in line with our proposed revision of the extra-pharmacological model (Figure 2) and as our cases demonstrate, one of the many lessons that may be learned from psychedelic experiences is how the human mind can be shaped by its environment. In essence, that what you put into your mind, you may well get out.

## Data availability statement

The original contributions presented in this study are included in the article/Supplementary material, further inquiries can be directed to the corresponding author.

## Ethics statement

The study was reviewed and approved by Douglas Mental Health University Institute and the Jewish General Hospital research ethics boards. The patients/participants provided their written informed consent to participate in this study. Written informed consent was obtained from the individual(s) for the publication of any potentially identifiable images or data included in this article.

## Author contributions

NG and KG: concept and design, drafting of the manuscript. KG, SR-D, and JT: administrative, technical, or material support. KG: supervision. All authors: acquisition, analysis, or interpretation of data, critical revision of the manuscript for important intellectual content.
