# Supplementary material for: Imprinting: expanding the extra-pharmacological model of psychedelic drug action to incorporate delayed influences of sets and settings
Source: Front Hum Neurosci. 2023 Jul 18;17:1200393. doi: 10.3389/fnhum.2023.1200393 (PMC10390742; doi:10.3389/fnhum.2023.1200393)
Supplement: Supplementary file 1 [file Table_1.DOCX]

Supplementary Material

**Imprinting: Expanding the Extra-Pharmacological Model of Psychedelic Drug Action to Incorporate Delayed Influences of Sets and Settings**

**Nicolas Garel, Julien Thibault Lévesque, Dasha A. Sandra, Justin Lessard-Wajcer, Elizaveta Solomonova, Michael Lifshitz, Stéphane Richard-Devantoy, Kyle T. Greenway***

***Correspondence:**

Kyle T. Greenway

[kyle.greenway@mail.mcgill.ca](mailto:kyle.greenway@mail.mcgill.ca)

**eTable 1:** Sociodemographic and baseline characteristics of ten patients receiving ketamine treatments for whom examples of imprinting were observed.

|  |  | **Total** | **SD or %** |
| --- | --- | --- | --- |
| **Age (mean + SD)** |  | 45.1 | 14.5 |
| **Female (number, %)** |  | 8 | 80% |
| **Race (number, %)** |  |  |  |
|  | White | 9 | 90% |
|  | Hispanic | 1 | 10% |
| **Work (number, %)** |  |  |  |
|  | Disability | 7 | 70% |
|  | Unemployed/Retired | 2 | 20% |
|  | Employed | 1 | 10% |
| **Relationship Status (number, %)** |  |  |  |
|  | Single | 6 | 60% |
|  | Divorced | 1 | 10% |
|  | Married/Cohabitating | 3 | 30% |
| **Education (number, %)** |  |  |  |
|  | ≤ High school | 1 | 10% |
|  | High school or college | 3 | 30% |
|  | University | 6 | 60% |
| **BMI (mean + SD)** |  | 29.5 | 11.6 |
| **DSM-5 Diagnosis (number, %)** |  |  |  |
|  | Bipolar II | 2 | 20% |
|  | Anxiety disorder | 5 | 50% |
|  | PTSD | 3 | 30% |
|  | Personality disorder | 7 | 70% |
|  | ADHD | 2 | 20% |
|  | Eating disorder | 1 | 10% |
|  | OCD | 1 | 10% |
| **Current episode (mean + SD)** |  |  |  |
|  | DM-TRD score | 16.0 | 1.76 |
|  | Other psychiatric diagnoses | 2.0 | 1.70 |
|  | Current episode duration (y) | 6.0 | 8.02 |
|  | Antidepressant trials | 3.5 | 1.08 |
| **Current and past episodes (number, %)** |  |  |  |
|  | Benzodiazepines | 6 | 60% |
|  | Antipsychotics or mood stabilizers | 7 | 70% |
|  | ECT | 2 | 20% |
|  | Hospitalization | 3 | 30% |
|  | Suicide attempt | 7 | 70% |
| **Physical health (number, %)** |  |  |  |
|  | Tobacco user | 1 | 10% |
|  | Cannabis user | 0 | 0% |
|  | Blood pressure medication | 2 | 20% |
|  | Diabetes melitus | 2 | 20% |
|  | Obstructive sleep apnea | 1 | 10% |
| **Patient Scores (mean + SD)** |  |  |  |
|  | BDI | 35.0 | 15.8 |
|  | SSI-C | 8.7 | 8.9 |
|  | SSI-W | 21.1 | 8.3 |
|  | PPP-VAS median physical pain | 28.3 | 15.9 |
|  | PPP-VAS median psychological pain | 57.7 | 23.0 |
|  | STAI-Trait | 55.8 | 7.9 |
|  | STAI-State | 52.5 | 12.2 |
|  | PSQI | 8.3 | 3.7 |
|  | QoL | 35.6 | 14.7 |
|  | STAR-P | 38.8 | 7.4 |
|  | CTQ | 43.9 | 24.8 |
|  | MODTAS | 49.3 | 26.8 |
| **Clinician Scores (mean + SD)** |  |  |  |
|  | MADRS | 28.3 | 8.3 |
|  | CGI Severity | 4.2 | 1.1 |
|  | CGI Suicidality | 2.6 | 1.0 |
|  | STAR-C | 38.0 | 8.3 |

BMI: Body Mass Index; DSM-5: Diagnostic and Statistical Manual of Mental Disorders, 5^th^ edition (American Psychiatric Association, 2013); PTSD: Post-Traumatic Stress Disorder; ADHD: Attention Deficit/Hyperactivity Disorder; OCD: Obsessive Compulsive Disorder; DM-TRD: Dutch Measure for quantification of treatment resistance in depression (Peeters et al., 2016); ECT: Electroconvulsive Therapy; BDI: Beck Depressive Inventory (Beck et al., 1996); SSI-C / SSI-W: Scale for Suicidal Ideation Current and Worst (Beck et al., 1979); PPP-VAS: Visual Analog Scale for psychological and physical pain (Jollant et al., 2019); STAI: State-Trait Anxiety Inventory (Spielberger et al., 1983); PSQI: Pittsburgh Sleep Quality Index (Buysse et al., 1989); QoL: Global Quality of Life Scale (Hyland and Sodergren, 1996); STAR-P / STAR-C: Scale to Assess Therapeutic Relationship Clinician and Patient Versions (McGuire-Snieckus et al., 2007); CTQ: Childhood Trauma Questionnaire (Bernstein et al., 1998); MODTAS: Modified Tellegen Absorption Scale (Jamieson, 2005); MADRS: Montgomory-Asberg-Depression-Scale (Davidson et al., 1986); CGI: Clinical Global Impression Scale Severity and suicidality (Guy, 1976)

**eTable 2:** Detailed transcripts from eight different patients receiving ketamine treatments providing examples of imprinting.

| **Example 1: Priming from a recently watched movie**  Participant: “To me, it felt apocalyptic, covered in … oh, and I also thought about this movie that we watched! Because I felt like I was in the movie.”  Interviewer: “Okay, what was the movie?”  Participant: “I’m trying to remember. It was a horrible movie. Just super boring. It was about this girl in a pod who ended up being in space. Have you heard of it?”  Interviewer: “I can’t guess it from that description. But it’s less important what the actual movie is. How did you relate to it?”  Patient: “I just felt like I was in a pod in space and I was thinking “Oh, it’s like that movie!” But then I was thinking ‘Oh, was it a movie or is this just my movie?” But then I remembered that it is a movie that I just watched.” |
| --- |
| **Example 2: Priming from a recently watched movie**  Interviewer: “Did you experience visual phenomena today?”  Patient: “Um, less so than the first two times, like, I did see myself as a fish.”  Interviewer: “Okay. Do you feel like a fish?”  Patient: “No, just like scales, golden shiny scales, I don’t know. Yesterday I watched ‘Luca’, which is a sea monster movie. Yeah, it’s like a boy, like, I mean, I don’t know if he’s a fish, he’s a sea monster, he has scales.”  Interviewer: “Oh. Is he the protagonist?”  Patient: “Yeah! I don’t know, I just think that’s why fish are on my mind.” |
| **Example 3: Priming from a recent nature experience**  The participant came back in Montreal from her cottage (a Camper Van) to receive her Ketamine infusion.  **Original transcript:**  Interviewer: “Qu’est-ce que vous avez expérimenté ?”  Participant : “Ben, dans ma tête j'ai revu l'environnement campagnard de mon chalet, les champs, des champs, c'est apaisant. Pis j'ai pris des photos dans ma tête. J'ai pris des photos parce que le foin a commencé à pousser, pis c’est beau, c’est haut, pis quand c'est en collines montantes. Ça, c'est un moment présent heureux.”  Interviewer : “Vous aviez l'air d'être pleinement dans ce moment-là pour le décrire de cette façon.”  Patient : “Je retrouve aussi les sons de mes petits oiseaux, quand j'étais couchée dans mon lit dans ma roulotte.”  Interviewer : “Il y en a combien, d'oiseaux, que vous entendez ?”  Patient : “Ah, c'est plein, beaucoup.”  Interviewer : “Ok. Est-ce qu'ils chantent tous de la même façon ?”  Patient : “Non. C'est plus le soir, le début de la nuit. [Mon mari] ronfle. Il faut que je l'accepte.”  Interviewer : “C'est un autre type d'oiseau, ça.”  **English translation:**  Interviewer: “What did you experience?”  Participant: “Well, in my head I saw the country environment again, the fields, the fields, it's soothing. And I took pictures in my head. I took pictures because the hay started to grow, and it's beautiful, it's high, and when it's in rising hills... That's a happy present moment.”  Interviewer: “You seemed to be fully in this moment to describe it that way.”  Patient: “I also hear the sounds of little birds, the same then when I was lying in my bed in my trailer.”  Interviewer: “How many of those, birds, did you hear?”  Patient: “Ah, it's a lot, a lot.”  Interviewer: “Okay, do they all sing the same way?”  Patient: “No. It's more in the evening, the beginning of the night. [My partner] snores. I have to accept that.”  Interviewer: “That's, a different kind of bird (laughing)” |
| **Example 4: Priming from a recent conversation**  **Original transcript (French):**  Interviewer: “Puis au niveau visuel, qu'est-ce que vous avez expérimenté pendant cette infusion?”   Patient: “C'est ça, je pensais induire un visuel plus de nature, mais non. Le cerveau se donne pas la peine de construire des choses aussi complexes (…) On était dans la couleur, les formes, beaucoup plus urbain. Il y a plus - il y avait de la structure, des volumes, des lignes droites.”  Interviewer : “Il y avait une atmosphère urbaine ?”  Patient : “Oui, comme des immeubles. Une sensation d’urbanisme.”  Interviewer : “Quand vous dites "Il y avait cette sensation d'urbanisme" ? – quelle sensation ?”  Patient : “C'est parce qu'on a parlé d'urbanisme dans l'auto en s'en venant, aussi.”  **English translation:**  Interviewer: “Then visually, what did you experience during this infusion?”  Patient: “That's it, I wanted to induce a more natural visual, but no. The brain doesn't bother to build such complex things (...).We were in colors, shapes, much more urban. There is more - there was structure, volumes, straight lines. An urban feel.”  Interviewer: “There was an urban atmosphere?”  Patient: “Yes, like buildings. An urban feeling.”  Interviewer: “When you say "There was this urban feel?" – what feeling?”  Patient: “That's because we talked a lot about urbanism in the car on the way there, too.” |
| **Example 5: Priming from a recent artistic experience**  **Original transcript (French):**  Patient : “Ça a été - ouf - un voyage incroyable.”  Interviewer : “C'est vrai ? Parlez-nous de ça.”  Patient : “Euh, c'était très axé sur les arts.”  Interviewer : “Qu'est-ce que vous voulez dire, sur les arts?”  Patient : “J'étais carrément au musée, encore, mais…dans les tableaux (…) ça a été une expérience artistique. J'ai fait de la peinture le weekend avant et j’étais allé au musé et là ça été le trip le plus total, j'en reviens pas. [...] C'était une expérience esthétique, artistique. J'étais dans l'art, j'étais dans la peinture. Ça m'a bien épaté!”  Interviewer : “Dans l'art, comme si vous étiez dans un tableau, par exemple?”  Patient : “Mais tout ça n'était pas figé, j'étais dans la couleur en mouvement, dans les formes en mouvement.”  **English translation:**  Patient: “It's been - wow - an incredible journey.”  Interviewer: “It has? Tell us about that.”  Patient: “Uh, it was very arts oriented.”  Interviewer: “What do you mean, arts oriented?”  Patient: “I was definitely in the museum, again, but...in the paintings (…) It was an artistic experience. I did some painting the weekend before and I went to the museum and it was a total trip, I can't believe it. [...] It was an aesthetic, artistic experience. I was in the art. It blew my mind!”  Interviewer: “In the art, as if you were in a painting, for example?”  Patient: “But it wasn't all frozen, I was in the color in movement, in the forms in movement.”  **Further description during post-treatment qualitative interview:**  **Original transcript (French):**  Patient : “J'ai l'impression que les évènements récents influencent. Comme par exemple le weekend avant la première séance, le premier lundi, j'avais complété une ou deux toiles. Fait que j'étais déjà dans les couleurs et l'art et ça été la continuité de ça. Le deuxième traitement, c'était pas le cas la veille, mais je me rappelle qu'avant le 3e traitement j'ai peint. Ah, avant le deuxième traitement, j'avais juste fait des fonds [de toile] et mes fonds sont toujours noir. Fait que je m'étais dit avant le troisième "non non, je vais travailler en coleur avant", pour ne pas être dans le noir comme pendant le deuxième traitement. Fait que j'ai l'impression que la mémoire à relativement court terme entre en ligne de compte.”  **English translation :**  Patient: “I feel like recent events influence. Like the weekend before the first session, the first Monday, I had completed one or two paintings. So I was already into colors and art and it was a continuation of that. The second treatment, it was not the case the day before, but I remember that before the third treatment I painted. Ah, before the second treatment, I had just done backgrounds and my backgrounds are always black. So before the third treatment I said to myself "no no, I'm going to work in color first", so that I wouldn't be in the dark like during the second treatment. So I feel like the relatively short term memory comes into play.” |
| **Example 6: Priming from recently watched videos**  **Original transcript (French):**  Interviewer : “Comment ça s’est passé ?”  Patient : “Beau…Vraiment beau.”  Interviewer : “Dit moi s’en plus ?”  Patient : “Tout bougeait, tout dansait.”  Interviewer : “Tu avais toi-même l’impression de danser ?”  Patient : “Non… Je voyais des gens danser devant moi… En fait je voyais une personne danser sur une scène… Elle dansait le ballet… C’était beau… J’assistais à un spectacle de danse…”  Interviewer : “Quelle sensation tu ressentais dans ton corps à ce moment ?”  Patient : “J’étais trop absorbé par la scène… C’était un spectacle en mouvement… J’adore la danse et je regarde toujours des vidéos de ballet sur mon téléphone cellulaire … C’était comme si j’étais dans un spectacle.”  **English translation:**  Interviewer: “How did it go?”  Patient: “Beautiful... Really beautiful.”  Interviewer: “Tell me more?”  Patient: “Everything was moving, everything was dancing.”  Interviewer: “Did you feel like you were dancing?”  Patient: “No... I saw people dancing in front of me... Actually I saw a person dancing on a stage... She was dancing the ballet... It was beautiful... I was watching a dance show...”  Interviewer: “What was the feeling in your body at that moment?”  Patient: “I was too absorbed in the scene... It was a moving performance... I love dance and I always watch ballet videos on my cell phone... It was like being in a show.” |
| **Example 7: Priming from recently watched videos**  **Original transcript (French):**  Interviewer : “Bonjour à nouveau.”  Patient : “Bonjour.”  Interviewer : “Alors, comment l’expérience s’est déroulée ?”  Patient : “C’était bizarre… J’étais dans un vortex… Un genre de vortex.”  Interviewer : “Un vortex ?”  Patient : “Oui, comme une spirale, et là je me suis ramassé dans un espace noir, et finalement j’étais comme sur un plateau de tournage.”  Interviewer : “Un plateau de tournage ? Vous voulez dire comme dans un film ?”  Patient : “Non… Il était en train de filmer le film… J’étais comme dans la mise en scène.”  Interviewer : “Vous étiez observateur ou vous étiez dans le film ?”  Patient : “J’étais à l’extérieur… Je regardais de haut; je voyais le plateau de tournage de tout en haut… Et il tournait des scènes de la servante écarlate.”  Interviewer : “De la servante écarlate ?”  Patient : “Oui… C’est une télésérie que je regarde présentement… J’essaye de moins regarder la télé, mais cette télésérie est trop bonne… Je me voyais comme de l’extérieur regarder le plateau de tournage qui tournait des scènes de la servante écarlate.”  **English translation:**  Interviewer: “Hi again.”  Patient: “Hello.”  Interviewer: “So how did the experience go?”  Patient: “It was weird... I was in a vortex... Kind of a vortex.”  Interviewer: “A vortex?”  Patient: “Yes, like a spiral, and then I was in a black space, and finally I was, like, on a film set.”  Interviewer: “A film set? You mean like in a movie?”  Patient: “No... They were filming the movie... I was, like, on the set.”  Interviewer: “Were you observing or were you in the movie?”  Patient: “I was on the outside... I was looking down; I could see the set from the top... And they were shooting scenes of Handmaid’s tale.”  Interviewer: “Of Handmaid’s tale?”  Patient: “Yeah... It's a TV show that I'm watching right now... I'm trying to watch less TV, but this TV show is too good... I saw myself from the outside looking down at the set filming scenes of the Handmaid’s tale.” |
| **Example 8: Priming from recently watched videos**  Interviewer: “What was your experience like?”  Patient: “It was really beautiful, but there was one strange thing. I kept seeing faces from that show my sister loves.”  Intervier: “Oh yeah?”  Patient: “Yeah! It’s very frustrating, I hate that show. You know the one, the historical drama. She always makes me watch it.” |

**References:**

AMERICAN PSYCHIATRIC ASSOCIATION 2013. *Diagnostic and Statistical Manual of Mental Disorders,* Washington, DC.

BECK, A. T., KOVACS, M. & WEISSMAN, A. 1979. Assessment of suicidal intention: the Scale for Suicide Ideation. *J Consult Clin Psychol,* 47**,** 343-52.

BECK, A. T., STEER, R. A., BALL, R. & RANIERI, W. 1996. Comparison of Beck Depression Inventories -IA and -II in psychiatric outpatients. *J Pers Assess,* 67**,** 588-97.

BERNSTEIN, D. P., FINK, L., HANDELSMAN, L. & FOOTE, J. 1998. Childhood trauma questionnaire. *Assessment of family violence: A handbook for researchers and practitioners.*

BUYSSE, D. J., REYNOLDS III, C. F., MONK, T. H., BERMAN, S. R. & KUPFER, D. J. 1989. The Pittsburgh Sleep Quality Index: a new instrument for psychiatric practice and research. *Psychiatry research,* 28**,** 193-213.

DAVIDSON, J., TURNBULL, C. D., STRICKLAND, R., MILLER, R. & GRAVES, K. 1986. The Montgomery‐Åsberg Depression Scale: reliability and validity. *Acta psychiatrica scandinavica,* 73**,** 544-548.

GUY, W. 1976. Clinical global impression (CGI). *ECDEU assessment manual for psychopharmacology,* 218**,** 222.

HYLAND, M. E. & SODERGREN, S. C. 1996. Development of a new type of global quality of life scale, and comparison of performance and preference for 12 global scales. *Quality of Life Research,* 5**,** 469-480.

JAMIESON, G. A. 2005. The modified Tellegen absorption scale: A clearer window on the structure and meaning of absorption. *Australian Journal of Clinical and Experimental Hypnosis,* 33**,** 119.

JOLLANT, F., VOEGELI, G., KORDSMEIER, N. C., CARBAJAL, J. M., RICHARD-DEVANTOY, S., TURECKI, G. & CÁCEDA, R. 2019. A visual analog scale to measure psychological and physical pain: A preliminary validation of the PPP-VAS in two independent samples of depressed patients. *Progress in Neuro-Psychopharmacology and Biological Psychiatry,* 90**,** 55-61.

MCGUIRE-SNIECKUS, R., MCCABE, R., CATTY, J., HANSSON, L. & PRIEBE, S. 2007. A new scale to assess the therapeutic relationship in community mental health care: STAR. *Psychological medicine,* 37**,** 85-95.

PEETERS, F. P., RUHE, H. G., WICHERS, M., ABIDI, L., KAUB, K., VAN DER LANDE, H. J., SPIJKER, J., HUIBERS, M. J. & SCHENE, A. H. 2016. The Dutch Measure for quantification of Treatment Resistance in Depression (DM-TRD): an extension of the Maudsley Staging Method. *J Affect Disord,* 205**,** 365-371.

SPIELBERGER, C., GORSUCH, R., LUSHENE, R., VAGG, P. & JACOBS, G. 1983. Manual for 22 the State-Trait Anxiety Inventory (STAI) Form Y. Palo Alto, CA Consulting 23 Psychologists Press. Inc.
